# Common practice patterns in the diagnosis and management of Vogt–Koyanagi–Harada syndrome: a survey study of uveitis specialists

**DOI:** 10.3389/fopht.2023.1217711

**Published:** 2023-07-07

**Authors:** Charlene H. Choo, Nisha R. Acharya, Jessica G. Shantha

**Affiliations:** F.I. Proctor Foundation, University of California San Francisco (UCSF), San Francisco, CA, United States

**Keywords:** Vogt–Koyanagi–Harada, VKH, practice patterns, survey study, ophthalmologist

## Abstract

**Introduction:**

Vogt–Koyanagi–Harada (VKH) syndrome is an inflammatory condition characterized by bilateral, granulomatous panuveitis with or without systemic manifestations, and accounts for up to 18% of referrals for panuveitis at tertiary centers in the United States of America. Despite ongoing research, there is limited evidence and no clear consensus on how to diagnose and treat patients with VKH, leading to variations in practice patterns among uveitis specialists.

**Methods:**

An anonymous, online survey was distributed to uveitis specialists in the American Uveitis Society (AUS). The survey included 21 questions that asked for non-identifiable demographics and covered topics such as preferred imaging modalities, treatment for the first episode of VKH, and perceived efficacy of immunomodulatory therapy (IMT).

**Results:**

A total of 104 surveys were included for analysis, representing a 38.4% response rate from the AUS listserv. A majority of respondents were uveitis fellowship trained and practiced in North America in an academic setting. Fluorescein angiography and enhanced depth imaging with optical coherence tomography were rated as the most consistently useful methods for the diagnosis of VKH. For treatment of acute initial-onset VKH, responses were divided between a preference for high-dose systemic corticosteroids with IMT (61.5%) and without IMT (37.5%). Methotrexate and mycophenolate mofetil were the most common IMTs to be used as first-line therapies, but adalimumab and infliximab were perceived to be the most effective for the treatment for VKH.

**Discussion:**

While there are some common trends in the practice patterns for the diagnosis and treatment of patients with VKH, there was no clear consensus on the topic of IMT. There was a slight preference among uveitis specialists to use both IMT and systemic corticosteroids for the first episode of acute VKH.

## Introduction

Vogt–Koyanagi–Harada (VKH) syndrome is a progressive inflammatory condition that presents with bilateral, granulomatous panuveitis and often involves the neurologic, auditory, and integumentary systems. The exact pathogenesis of VKH is not known but likely involves a T-cell-mediated immune response against antigens of melanocytes in genetically susceptible individuals (1). VKH is more prevalent in Asians in countries such as Japan and China and in populations with dark skin pigmentation such as Hispanics and Native Americans, but is a common cause of panuveitis in the United States of America, accounting for up to 18% of referrals at tertiary centers (2).

The diagnosis of VKH is challenging due to a wide array of ocular and systemic manifestations that occur at different stages of the disease and a lack of conclusive findings in clinical exams, laboratory investigations, or imagings. The revised diagnostic criteria by the American Uveitis Society (AUS) determined early and late manifestations of VKH, such as subretinal fluid or detachment and choroidal inflammation in the early stage and depigmentation of the posterior segment (“sunset glow fundus”) and perilimbal vitiligo (Sugiura’s sign) in the late stage (3). Separate criteria for early- and late-stage VKH were recently developed by the Standardization of Uveitis Nomenclature (SUN) Working Group, highlighting the distinct ophthalmic and systemic manifestations that characterize disease progression (4). Newer imaging modalities that improve visualization of the choroid, such as enhanced depth imaging with optical coherence tomography (EDI-OCT) and indocyanine green angiography (ICGA), were incorporated into recently published diagnostic criteria by Yang and colleagues as well as by Herbort and colleagues (5, 6). However, it is unclear whether these new diagnostic criteria influenced how often ophthalmologists use newer imaging modalities like EDI-OCT or ICGA to diagnose and manage VKH.

The treatment regimen for acute initial-onset VKH traditionally consisted of high-dose systemic corticosteroids with the addition of immunomodulatory therapy (IMT) if the inflammation persisted or recurred. Not only are there no clear guidelines for the route of administration or duration of treatment with systemic corticosteroids, but there is also growing evidence that corticosteroid monotherapy may be insufficient to prevent recurrent or chronic disease in patients with VKH. A systematic review of patients with acute initial-onset VKH found that 44% had recurrence and 59% developed “sunset glow fundus,” or depigmentation of the fundus, despite treatment with high-dose systemic corticosteroids (7). On the other hand, early initiation of IMT has been shown to improve visual outcomes and remission rates in patients with acute VKH in a few small studies (8–10).

In the absence of any large randomized clinical trials, there is little information to guide ophthalmologists on the treatment of acute VKH, particularly regarding the use of corticosteroid-sparing IMT. This has likely led to large variations in how VKH is managed. The purpose of this survey study is to provide a cross-sectional view of the current practice patterns and identify areas of divergence in the diagnosis and management of VKH.

## Methods

This study was deemed exempt by the University of California San Francisco (UCSF) institutional review board. An anonymous survey was developed, and responses were collected using the web-based UCSF secure Qualtrics^XM^. The survey consisted of a total of 21 questions. Topics of interest included non-identifiable demographic characteristics, ophthalmic imaging trends, and treatment patterns and preferences in the management of VKH. Question types included multiple choice questions, free-text responses, a sliding scale ranging from 0 to 100, and a Likert scale of frequency (never, rarely, sometimes, frequently, always). Acute initial-onset VKH was defined as a first episode of acute bilateral panuveitis with subretinal detachments.

The survey was distributed in July of 2022 by email to the AUS listserv, which currently has 271 active members. The AUS is a select group of uveitis specialists from around the world. Membership requires two letters of recommendation and one of the following criteria: completion of an Association of University Professors of Ophthalmology (AUPO)-compliant uveitis fellowship, at least 30% of professional time dedicated to caring for uveitis patients and/or ocular immunology research, or publication of two peer-reviewed papers in the field of uveitis or ocular immunology as the first or second author.

Surveys that were 70% or more complete were included in the study. Statistical analysis was performed using the R program v.3.6.2 for Linux (R Foundation for Statistical Computing, Vienna, Austria). Data were summarized with descriptive statistics using means, medians, standard deviations (SD), and interquartile ranges (IQR) when appropriate. A *p* value of ≤ 0.05 was considered statistically significant. Paired *t*-tests and ANOVA tests were used to compare continuous variables with a normal distribution. A chi-squared test was used for categorical variables.

## Results

### Physician demographics and characteristics

Of the 271 AUS listserv members, 104 unique surveys were completed, representing a 38.4% response rate from the AUS listserv. A majority of respondents were uveitis fellowship trained (94.1%), had been in clinical practice for 5 years or more after their fellowship (79.6%), and were working in North America (62.1%) in an academic setting (83.7%) (**Table 1**). Corticosteroid-sparing IMT was prescribed by the ophthalmologist or rheumatologist, or co-managed in 68.3%, 14.4%, and 17.3% of cases, respectively.

**Table 1:** Demographic characteristics of survey respondents.

Characteristic	*n* (%)
Fellowship training	
Uveitis	40 (39.2)
Uveitis and medical/surgical retina	47 (46.1)
Uveitis and other	9 (8.8)
Other	6 (5.9)
Years in practice	
More than 10 years	58 (56.3)
5 to 10 years	24 (23.3)
0 to 5 years	21 (20.4)
Location	
North America	64 (62.1)
Asia	11 (10.7)
South America	10 (9.7)
Europe	6 (5.8)
Middle East	5 (4.9)
Other (Oceania, Africa, Central America)	7 (6.8)
Practice setting	
University or academic center	60 (57.7)
Hybrid (private practice and academic)	27 (26.0)
Private practice	10 (9.6)
Other	7 (6.7)

### Disease characteristics and ophthalmic imaging

**Figure 1:**
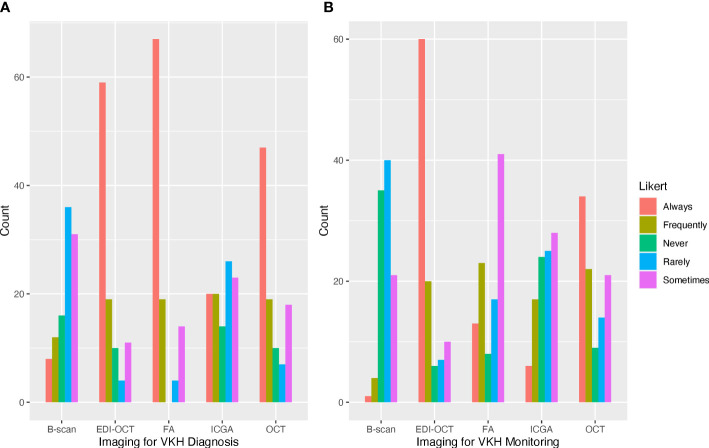
Frequently used ophthalmic imaging techniques for the diagnosis and management of Vogt–Koyanagi–Harada (VKH). **(A)** Fluorescein angiography and optical coherence tomography (OCT) with and without enhanced depth imaging (EDI) were frequently reported as always used for the diagnosis of VKH. **(B)** OCT with EDI was most frequently used for the management of VKH.

In the last year, most respondents managed 1–5 cases (60.6%) or 5–10 cases (26.0%) of acute VKH, while fewer respondents managed 10–15 cases (5.8%) or 15–20 cases (3.5%). Three respondents (2.9%) managed no cases of acute VKH, and one respondent (1.0%) managed more than 20 cases. Respondents reported an average recurrence rate of 49.7% (SD = 29.2) for their patients with VKH. For the diagnosis of acute VKH, a majority of respondents rated FA (64.4%) and EDI-OCT (64.4%) as always useful for the diagnosis of VKH. Fewer respondents rated OCT without EDI (46.5%), ICGA (19.4%), or B-scan ultrasonography (7.8%) as always useful in the diagnosis of VKH (**Figure 1A**). For disease monitoring, most respondents reported EDI-OCT as always useful (58.2%), followed by OCT without EDI (34.0%), FA (12.7%), ICGA (11.9%), and B-scan ultrasonography (0.1%) (**Figure 1B**). Optical coherence tomography angiography was entered as free text by nine (8.6%) respondents as useful in evaluating choroidal flow voids and neovascularization.

### Treatment patterns for acute VKH

For a patient presenting with their first episode of VKH with bilateral panuveitis and subretinal detachments, 61.5% of respondents selected high-dose systemic corticosteroids with IMT as the treatment of choice. In conjunction with IMT, the most frequently used form of corticosteroid was oral (40.6%), followed by oral and intravenous (IV) (28.1%), and IV corticosteroids (14.1%). Corticosteroid monotherapy was preferred as the first-line treatment by 37.5% of respondents, for which oral corticosteroids (51.3%) were most commonly used, followed by various combinations of IV, local, and/or oral corticosteroids (33.3%), and IV corticosteroid monotherapy (15.4%). One respondent (1.0%) preferred IMT without systemic corticosteroids for the treatment of acute VKH.

High-dose systemic corticosteroids were most commonly prescribed for a period of 3 to 6 months (43.2%), followed by 3 months or less (31.6%), 6 to 12 months (22.1%), and more than 12 months (3.2%). A majority of ophthalmologists who preferred high-dose systemic corticosteroids with IMT as the first-line treatment for acute initial-onset VKH reported treating with systemic corticosteroids for 6 months or less (82.2%). Among ophthalmologists who preferred corticosteroid monotherapy, 60.6% reported treating for 6 months or less while 39.4% reported treating for more than 6 months. The treatment of choice for acute initial-onset VKH was found to be significantly associated with the duration of treatment with systemic corticosteroids (*p* = 0.039).

Factors that were rated as always impacting the clinical decision to start IMT included the desire for a shorter duration of treatment with systemic corticosteroids (30.1%), the presence of subretinal fluid (27.9%), and systemic features of VKH (21.2%). Fewer respondents rated anterior segment inflammation with posterior synechiae (16.3%) and initial visual acuity (15.4%) as always impacting the clinical decision to start IMT (**Table 2**).

**Table 2:** Factors impacting clinical decision to start corticosteroid-sparing immunosuppression, *n* (%).

Frequency	Initial visual acuity	Anterior segment inflammation and PS	Subretinal fluid	Systemic features of VKH	Desire for shorter treatment with systemic CS
Always	16 (15.4)	17 (16.3)	29 (27.9)	22 (21.2)	31 (30.1)
Frequently	28 (26.9)	34 (32.7)	25 (24.0)	31 (29.8)	37 (35.9)
Sometimes	26 (25.0)	19 (18.3)	23 (22.1)	20 (19.2)	21 (20.4)
Rarely	16 (15.4)	17 (16.3)	12 (11.5)	15 (14.4)	9 (8.7)
Never	18 (17.3)	17 (16.3)	15 (14.4)	16 (15.4)	5 (4.9)

PS, posterior synechiae; VKH, Vogt–Koyanagi–Harada; CS, corticosteroid.

The most preferred first-line IMT by class was antimetabolites (53.9%), followed by antimetabolites with biologics (30.4%), cyclosporine with antimetabolites and/or biologics (9.5%), and other (3.9%). The majority of respondents (69.6%) had more than one IMT that they would prescribe as the first-line treatment. Of these, methotrexate and mycophenolate mofetil were the most frequently selected (67.6% and 66.7%, respectively), followed by adalimumab (35.3%), azathioprine (22.5%), infliximab (11%), and cyclosporine (10.8%).

### Perceived medication effectiveness

The survey included questions about the ophthalmologists’ beliefs on the efficacy of VKH treatment, rated from 0% to 100%. Systemic corticosteroid monotherapy was rated as 37.5% effective in preventing VKH recurrence and 34.1% effective in preventing “sunset glow fundus,” or depigmentation of the choroid. These results were found to be significantly different from beliefs about the combination of corticosteroids with IMT, which respondents rated as 76.1% effective in preventing future flares and 67.4% effective in preventing “sunset glow fundus” (*p* < 0.001 and *p* < 0.001).

**Figure 2:**
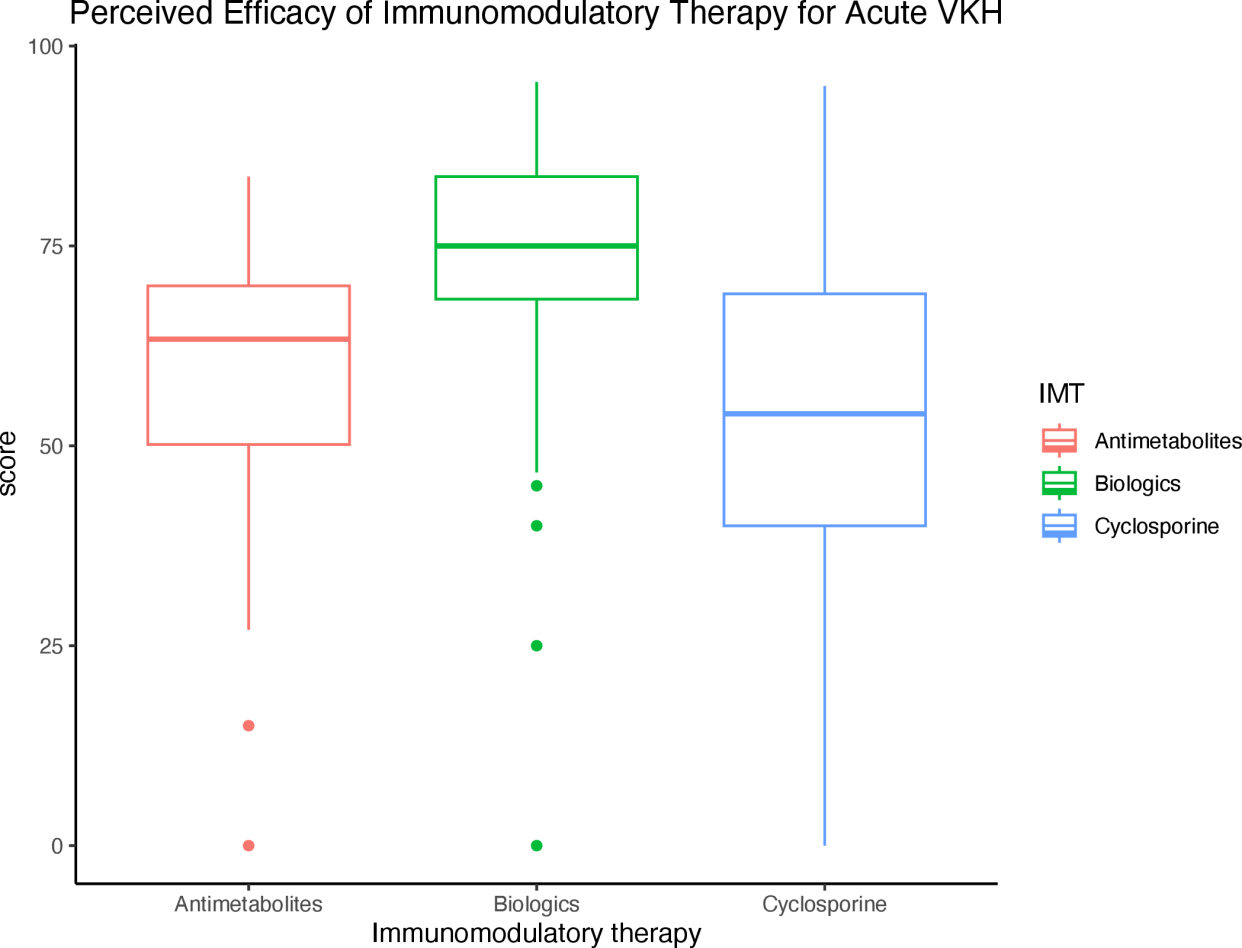
Perceived efficacy of immunomodulatory therapy (IMT) by class for the treatment of acute VKH Boxplot diagram demonstrates the perceived efficacy of IMTs by class for the treatment of VKH, of which biologics were rated as the most effective.

For individual IMT, infliximab (78.8%), adalimumab (77.0%), and mycophenolate mofetil (65.8%) were rated as the most effective in treating VKH. When grouped by IMT class, biologics were rated as the most effective (73.4%), followed by antimetabolites (59.7%), and cyclosporine (51.1%) (**Table 3**). The difference in perceived efficacy was found to be significantly different among the three classes of IMT, with the greatest difference between the perceived efficacy of biologics and that of cyclosporine or antimetabolites (*p* < 0.001 and *p* < 0.001, **Figure 2**).

**Table 3:** Perceived efficacy of immunosuppression for treatment of VKH disease.

Immunosuppression	Perceived efficacy (%)	
	Mean	SD
*Antimetabolites*	
Methotrexate	58.2	20.2
Mycophenolate mofetil	65.8	19.0
Azathioprine	56.8	19.9
*IMT type Calcineurin Inhibitor*	
Cyclosporine	51.1	22.3
*Biologics*	
Adalimumab	77.0	16.0
Infliximab	78.8	17.8
Tocilizumab	59.0	25.4

VKH, Vogt–Koyanagi–Harada; SD, standard deviation.

## Discussion

The results of our study indicate that there is a lack of consensus among uveitis specialists on the treatment of VKH, especially regarding the use of IMT for acute VKH.

Uveitis specialists in the study reported a recurrence of almost 50% in their patients with VKH, which is similar to what has been reported in the literature (7). About 60% of uveitis specialists preferred and prescribed IMT in addition to systemic corticosteroids for the treatment of acute initial-onset VKH and believed that IMT with corticosteroid taper was significantly more effective in preventing recurrence and/or development of “sunset glow fundus” in patients with VKH than monotherapy with systemic corticosteroids. An interesting finding was that the preference to start IMT as the first-line therapy was significantly associated with a shorter duration of treatment with systemic corticosteroids. This can be explained by the survey result that showed that a desire to shorten treatment with systemic corticosteroids was a consistent factor affecting the decision to start IMT. Another explanation is that uveitis specialists who prefer first-line IMT tend to start tapering systemic corticosteroids at an earlier time point, perhaps after a few months when IMTs such as methotrexate or mycophenolate mofetil reach therapeutic doses. It is also possible that the addition of first-line IMT is associated with quicker inflammation control, leading to corticosteroid-sparing effects.

A majority of uveitis specialists reported using methotrexate and mycophenolate mofetil as the first-line IMT for acute VKH. In another survey study that included uveitis specialists from the AUS listserv, methotrexate was the most commonly used IMT for all subgroups of NIU, followed by mycophenolate mofetil for posterior and panuveitis (11). This is consistent with results from the First-line Antimetabolites as Steroid-sparing Treatment (FAST) uveitis clinical trial, which reported similar efficacy levels of methotrexate and mycophenolate in patients with NIU, but better efficacy levels of methotrexate in patients with posterior uveitis or panuveitis (12). A subanalysis of the FAST trial also found that a majority of patients with subretinal detachments associated with VKH achieved resolution of inflammation after treatment with systemic corticosteroids with methotrexate or mycophenolate mofetil, but did not find a difference in efficacy between the two agents (13). There is limited research on the use IMT as the first-line treatment for acute VKH, but a small prospective study also demonstrated better visual acuity and decreased recurrence of acute VKH after treatment with mycophenolate mofetil and systemic corticosteroids than those treated with corticosteroid monotherapy (14).

Biologics including adalimumab and infliximab were rated as most effective for the treatment of VKH but were selected by only 35.0% and 11.0% of respondents as first-line IMT, respectively. Reasons for prescribing antimetabolites instead of biologics likely include cost, safety, and ease of use of oral formulations (11). Among biologics, adalimumab has the most robust clinical evidence and was approved by the United States Food and Drug Administration (FDA) for non-anterior NIU in 2016 (15). Most of the studies evaluating adalimumab for the treatment of VKH are retrospective and involve patients with chronic VKH who previously failed therapy with systemic corticosteroids or other IMT, making it difficult to determine its efficacy as a first-line IMT. A study in Japan reported that 78.6% of patients with VKH, most of whom had late-stage disease, had a relapse during treatment with adalimumab, but found that a majority of patients had remission of disease with a combination treatment of methotrexate and adalimumab (16).

Multimodality imaging has emerged as a key tool for the diagnosis and management of uveitis. In VKH, the choroid is the predominant site of inflammation and characteristic features have been identified on various imaging modalities, such as FA, ICGA, and OCT with or without EDI. In our survey study, we found that uveitis specialists commonly use FA and OCT with or without EDI for the diagnosis of VKH. For disease monitoring, OCT with and without EDI were rated as more useful than FA and ICGA. Studies have shown that EDI-OCT and ICGA were useful in detecting subclinical recurrence in the choroid, helping to prevent chronic disease in patients with VKH (17, 18). However, not all practices are equipped with these imaging modalities and FA and ICGA are more invasive and time consuming, which may explain why ICGA was rated as less useful than conventional imaging tools and FA was not rated as useful in the disease monitoring phase. Evaluation of the choroid has become increasingly important in VKH since characteristic findings on imaging have come to serve as biomarkers of disease activity. More studies are needed to determine the utility of various imaging techniques in the disease-monitoring phase of VKH.

This study has several limitations. The survey was distributed through the AUS listserv, which includes uveitis specialists who mostly practice in an academic setting and limits generalizability to all practicing ophthalmologists. Respondents may have varying levels of recall with regard to diagnosing and treating patients with VKH. In addition, the question-and-answer choices in the survey encompassed a select number of ocular manifestations, image findings, and treatment options for VKH that the authors determined as most important for the purpose for the study. Thus, results of the study may not represent the entire spectrum of practice patterns.

## Data availability statement

The original contributions presented in the study are included in the article/supplementary material. Further inquiries can be directed to the corresponding author.

## Author contributions

JS and NA contributed to the conception and design of the study and data collection. CC performed the statistical analysis. CC, JS, and NA contributed to writing the manuscript. All authors contributed to the article and approved the submitted version.
